# Correlation of psoas major muscle morphology with function and clinical symptoms in patients with symptomatic multilevel lumbar spinal stenosis

**DOI:** 10.1186/s13018-023-03596-w

**Published:** 2023-02-15

**Authors:** Xiaofei Hou, Hailiang Hu, Chao Kong, Yongjin Li, Sitao Zhang, Wei Wang, Shibao Lu

**Affiliations:** 1 Department of Orthopaedics, Xuanwu Hospital, Capital Medical University, 45 Changchun Street, Xicheng District, Beijing, 100053 People’s Republic of China; 2 China National Clinical Research Center for Geriatric Disorders, Xuanwu Hospital, Capital Medical University, Beijing, People’s Republic of China

**Keywords:** Symptomatic multilevel lumbar spinal stenosis, Psoas major muscle, Function, Clinical symptoms

## Abstract

**Objective:**

This study was performed to quantify the morphological characteristics of the psoas major muscle in patients with symptomatic multilevel degenerative lumbar spinal stenosis (SMLSS) and assess the correlations of these morphological characteristics with function and clinical symptoms.

**Methods:**

One hundred fourteen patients diagnosed with SMLSS (≥ 3 segments) were included. The patients’ presenting symptoms were assessed with the Oswestry Disability Index (ODI), and visual analogue scale (VAS) scores were recorded. The morphology of the psoas major was evaluated at the L3/4 intervertebral disc level in three ways: by measuring (i) the psoas muscle mass index (PMI); (ii) the mean muscle attenuation (Hounsfield units, HU); and (iii) the morphologic change of the psoas major (mean ratios of the short axis to the long axis of the bilateral psoas major).

**Results:**

Men had a higher PMI than women (*p* = 0.001). Patients with severe disability had a significantly lower PMI (*p* = 0.002) and muscle attenuation (*p* = 0.001). The PMI and muscle attenuation were significantly higher in the patients with no or mild back pain (both *p* < 0.001). In the univariable and multivariable analyses, a greater HU value was associated with a higher functional status as assessed by the ODI (*p* = 0.002), and a higher PMI was associated with less severe back pain as measured by the VAS score (*p* < 0.001).

**Conclusion:**

This study showed that muscle attenuation of psoas major positively correlated with the functional status and PMI negatively correlated with low back pain severity in patients diagnosed with SMLSS. Future prospective studies are needed to evaluate whether improvement in such muscle parameters through physiotherapy programs can alleviate the clinical symptoms and improve the functional status of patients with SMLSS.

## Introduction

Degenerative lumbar spinal stenosis (LSS) is a narrowing of the spinal canal that results from loss of height of the intervertebral disc, hypertrophy of the facet joints, and thickening of the ligamentum flavum that compromises the neural structures at one or several levels of the lumbar spine [1]. LSS is commonly associated with aging and progressive degenerative processes of the spine [2]. Patients often develop symptoms such as intermittent claudication, low back pain, and leg pain and/or weakness [3, 4]. The results of a long-term follow-up study showed that only a portion of patients had aggravated symptoms, and the underlying mechanism was unclear [5–7]. Identifying the factors that influence function and symptoms in patients with LSS is of great clinical significance for predicting the prognosis and guiding treatment [8].

The psoas major has been considered to play a critical role in maintaining the physiological functions of the lumbar spine [9]. In patients with LSS (mainly single-level LSS), the psoas cross-sectional area (CSA) was significantly larger in the high than low functional performance group [10, 11]. The researchers speculated that this might be because the psoas major can serve as a spine stabilizer. In contrast to single-level LSS, symptomatic multilevel LSS (SMLSS) is usually associated with extensive spinal degeneration, such as osteophyte formation, spondylolisthesis, which indicate different biomechanical characteristics [12]. There hasn't yet been any research on how the psoas major muscle's morphology links to clinical symptoms and function in individuals with SMLSS. Patients with SMLSS are typically elderly and have severe clinical symptoms [13]. The risks of multilevel spinal decompression and fusion surgery for SMLSS are significant, and the prognosis for surgical treatment is still debatable [14, 15]. Therefore, understanding how psoas major muscle corresponds with the functional status and symptoms of patients with SMLSS is essential for developing new treatments (such as physiotherapy programs).

The primary purpose of the present study was to investigate the association of the psoas major muscle size, density, and shape with the functional status and visual analogue scale (VAS) scores for the back and lower extremities in patients with SMLSS. We hypothesized that higher psoas major muscle CSA (represented by the psoas major index, PMI), muscle density (represented by muscle attenuation on CT images) and morphologic change of the psoas muscle (MPM) would be associated with less pain severity and higher functional status in patients with SMLSS.

## Methods

### Participants

The records of patients who were admitted to our hospital because of SMLSS (≥ 3 segments) from January 2019 to December 2020 were screened for study eligibility. The diagnostic criteria for SMLSS were the presence of intermittent claudication, back and/or leg pain, and imaging features of multilevel LSS on lumbar magnetic resonance imaging (MRI) or computed tomography (CT). We excluded patients with spinal deformity (scoliosis of > 10° and/or a sagittal vertical axis of > 50 mm), patients without available preoperative CT or MRI examinations within 2 years of surgery, patients with a history of lumbar surgery, and patients with cachexia due to infectious diseases, cancer, myopathies, or dyskinesia. This study was approved by the institutional review board following the declaration of Helsinki principles at our hospital (No. 2018014). As a retrospective study and the data analysis were performed anonymously.

One hundred fourteen consecutive patients were selected, and the following basic data were collected for analysis: age, sex, body mass index (BMI), comorbidities, number of vertebral levels involved, and symptom duration. Functional performance was evaluated using the Oswestry Disability Index (ODI) score, and back and leg pain severity was evaluated using VAS scores.

### Analytical morphometrics

**Fig. 1:**
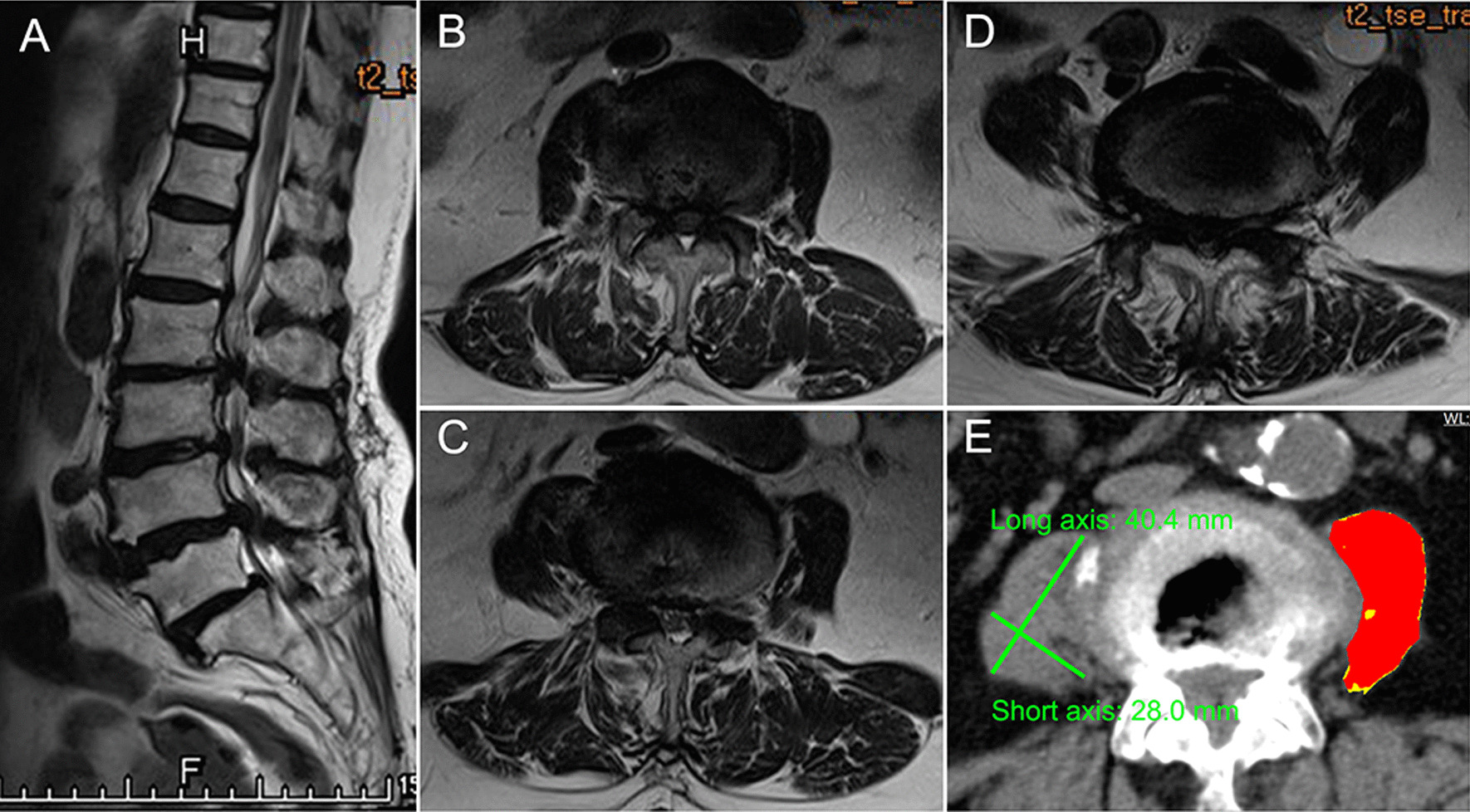
Imaging data of a 79-year-old woman with symptomatic multilevel lumbar spinal stenosis and measurement schematics of the psoas major at the L3/4 intervertebral disc level on an axial CT slice. **A** Sagittal MRI demonstrated multilevel lumbar spinal stenosis at L2–5. **B**–**D** Axis MRI depicted spinal stenosis at L2/3, L3/4 and L4/5. **E** The red area shows the psoas muscle area with CT values (pixel intensity) of − 29 to + 150. The mean muscle attenuation is the average CT value of the red regions. The MPM score is the ratio of the short axis to the long axis of the psoas major. Abbreviations: CT, computed tomography; MRI, magnetic resonance imaging; MPM, morphological change in the psoas major

A single axial CT image corresponding to the L3/4 intervertebral disc level was selected for radiological measurement. The Advanced Visualization Workspace 2.0 (Neusoft, Shenyang, Liaoning, China) was used to quantify skeletal muscles within predefined validated boundaries of − 29 to + 150 Hounsfield units (HU) to exclude vasculature, bony structures, and areas of intramuscular fatty infiltration. The total CSA of the psoas major was determined in semiautomated fashion with manual tracing of the bilateral psoas major borders. The psoas major muscle mass index (PMI) was calculated by normalizing the CSA for height (cm^2^/m^2^) (Fig. 1) [16]. The mean muscle attenuation (HU value) was calculated by averaging the CT values (pixel intensity) of the bilateral psoas major regions outlined on the images (Fig. 1) [17]. The morphologic change of the psoas muscle (MPM) was measured using previously reported methods [18]. In brief, the MPM score was defined as the mean of the respective ratios of the short axis to the long axis of the right and left psoas muscles (Fig. 1).

### Statistics

SPSS 22.0 statistical software (IBM Corp., Armonk, NY, USA) was used for statistical analysis. Measurement data are described as mean ± standard deviation. Categorical data are described as frequency (percentage). Continuous data were tested for normality. If the data were normally distributed and the variances between the two groups were equal, the two-sided Student’s t-test was applied to evaluate differences between groups; otherwise, the nonparametric Wilcoxon rank-sum test was considered. For ordered categorical data, the nonparametric Wilcoxon rank-sum test was used. For the factors that may affect the ODI, VAS score, and muscle parameters, univariate linear regression was performed, and the factors with a *p* value of < 0.05 in the univariate analysis were included in multivariate linear regression. A *p* value of < 0.05 was considered statistically significant.

## Results

### Descriptive data

In total, 114 patients were identified and entered into the database. The study group comprised 41 men and 73 women with a mean age of 69.9 ± 9.7 years. Major comorbidities were hypertension (63.2%), diabetes (30.7%), and cardiovascular disease (17.5%). A total of 12.3% of patients had a history of spinal disease other than lumbar disease. The duration of lower extremity pain ranged from 3 to 240 months, with a mean of 36.9 ± 55.9 months; back pain had been present for much longer (mean, 77.1 ± 79.9 months).

In the functional status and pain severity assessment, the mean ODI score was 54% ± 14%, the mean VAS score for back pain was 4.5 ± 2.4, and the mean VAS score for leg pain was 4.4 ± 2.8. Table 1 shows the detailed demographic data of the patients.

**Table 1:** Summary of descriptive features

Variable	All patients (*n* = 114)
*Demographics*	
Age (years)	
Mean ± SD	69.9 ± 9.7
*Gender*	
Female	73 (64%)
Male	41 (36%)
*BMI (kg/m*^*2*^*) (Mean ± SD)*	
Total	26.6 ± 4.0
Female	26.8 ± 3.9
Male	26.2 ± 4.2
*Comorbidity*	
Smoking	17 (15.0%)
Alcohol	16 (11.4%)
Diabetes	35 (30.7%)
Hypertension	72 (63.2%)
Cerebrovascular disease	14 (12.3%)
Cardiovascular disease	20 (17.5%)
Spinal disease	14 (12.3%)
*Clinical assessment*	
Symptom duration (month)	
Back pain duration	77.1 ± 79.9
Leg pain duration	36.9 ± 55.9
ODI	0.54 ± 0.14
VAS (back pain)	4.5 ± 2.4
VAS (leg pain)	4.4 ± 2.8

*SD* standard deviation; *BMI* body mass index; *ODI* Oswestry Disability Index; *VAS* visual analogue scale

### Morphometric results

Table 2 shows descriptive information regarding the morphometric measurements for all patients. Men had a significantly higher PMI than women (6.51 ± 1.31 vs. 5.17 ± 1.30, respectively; *p* < 0.001). There was no significant difference in the HU value or MPM between men and women.

**Table 2:** Morphometric measurements of psoas major in patients with SMLSS

Measurements	All patients (*n* = 114)	Female (*n* = 73)	Male (*n* = 41)	*P*-value
PMI	5.62 ± 1.45	5.17 ± 1.30	6.51 ± 1.31	**< 0.001**
HU	36.52 ± 5.35	35.77 ± 4.74	37.84 ± 6.15	0.104
MPM	0.59 ± 0.11	0.58 ± 0.11	0.59 ± 0.11	0.910

Bold indicates statistical significance

*SMLSS* symptomatic multilevel lumbar spinal stenosis; *PMI* psoas major muscle mass index; *HU* Hounsfield units; *MPM* morphological change in the psoas major

The reliability of morphological measurements of the psoas muscle used in this study was determined to be excellent. The inter-rater reliability coefficients were high (≥ 0.93).

### Patient characteristics and muscle parameters in different ODI performance and pain severity groups

The patients were divided into different groups according to their ODI scores (≤ 42, moderate disability; > 42, severe disability) [19] and VAS scores (≤ 3, no or mild pain; > 3, moderate or severe pain) [20] for comparison of the clinical characteristics and muscle parameters between the groups.

Patients with severe disability had a significantly lower PMI (*p* = 0.002) and HU value (*p* = 0.001) than patients with moderate disability. The duration of back pain was longer in patients with severe disability (*p* = 0.037). No significant differences in age, sex, BMI, leg pain duration, number of levels involved, or MPM were found between the two performance groups.

The PMI and HU value were significantly higher in the patients with no or mild back pain than in those with moderate or severe back pain (both *p* < 0.001). The back pain duration was longer in patients with moderate or severe pain (*p* = 0.004).

No significant differences in the patient characteristics or muscle parameters were found between different leg pain severity groups. Table 3 shows the detailed statistical data of these analyses.

**Table 3:** Clinical characteristics and morphometric parameters in different ODI performance and pain severity groups

	Functional disability			Pain intensity (VAS for back)			Pain intensity (VAS for leg)		
	ODI ≤ 42 *N* = 14	ODI > 42 *N* = 100	*p* value	VAS ≤ 3 *N* = 26	VAS > 3 *N* = 88	*p* value	VAS ≤ 3 *N* = 33	VAS > 3 *N* = 81	*p* value
*Gender*									
Male	7	34	0.243	13	28	0.09	16	25	0.075
Female	7	66		13	60		17	56	
Age (years) (mean ± SD)	70.1 (8.0)	70.1 (9.2)	0.985	70.0 (6.8)	70.1 (9.6)	0.759	71.1 (9.0)	69.7 (9.0)	0.305
BMI (kg/m^2^) (mean ± SD)	26.71 (4.55)	26.56 (3.99)	0.896	27.07 (3.62)	26.44 (4.16)	0.313	26.79 (3.87)	26.49 (4.12)	0.722
Back pain duration	35.5 (59.2)	89.9 (80.9)	**0.037**	60.0 (88.6)	82.1 (77.0)	**0.004**	74.9 (71.2)	78.0 (83.6)	0.851
Leg pain duration	17.5 (39.7)	26.0 (58.4)	0.165	43.9 (65.8)	34.9 (52.9)	0.235	34.6 (59.5)	37.9 (54.7)	0.305
Number of levels involved (mean ± SD)	3.14 (0.36)	3.27 (0.47)	0.332	3.19 (0.49)	3.27 (0.45)	0.337	3.30 (0.47)	3.23 (0.45)	0.389
PMI	6.75 (2.18)	5.51 (1.26)	**0.002**	6.93 (1.52)	5.28 (1.20)	**< 0.001**	5.60 (1.52)	5.68 (1.43)	0.802
HU	40.88 (5.07)	35.91 (5.12)	**0.001**	40.21 (5.25)	35.43 (4.90)	**< 0.001**	35.86 (6.88)	36.78 (4.62)	0.405
MPM	0.58 (0.10)	0.59 (0.11)	0.735	0.60 (0.09)	0.59 (0.11)	0.659	0.57 (0.09)	0.60 (0.11)	0.152

Bold indicates statistical significance

*ODI* Oswestry Disability Index; *VAS* visual analogue scale; *SD* standard deviation; *BMI* body mass index; *PMI* psoas major muscle mass index; *HU* Hounsfield units; *MPM* morphological change in the psoas major

### Association of patient characteristics and muscle parameters with function and pain severity

Age, BMI, symptom duration, and number of levels involved were not associated with function as measured with the ODI in the univariate analyses (Table 4). Patients with more severe functional disability had a lower PMI (*p* < 0.001) and HU value (*p* < 0.001). These muscle parameters and sex were entered into the multivariable regression models. The results show that muscle attenuation could negatively influence function (*p* = 0.002) and explained 30.4% of the variance in the ODI score.

**Table 4:** Association of patient characteristics and muscle parameters with function as measured with ODI

Subject characters	Univariable analysis		Multivariable analysis	
	Coefficient, 95% CI	*p* value	Coefficient, 95% CI	*p* value
Gender	− 0.059 (− 0.112, − 0.006)	**0.029**	− 0.023 (− 0.078, 0.032)	0.401
Age	− 0.001 (− 0.004, 0.002)	0.628		
BMI	0.005 (− 0.001, 0.011)	0.128		
Back pain duration	0.0003 (0.000, 0.001)	0.057		
Leg pain duration	0.0002 (− 0.002, 0.001)	0.333		
Number of levels involved	0.024 (− 0.033, 0.080)	0.411		
PMI	− 0.031 (− 0.048, − 0.015)	**< 0.001**	− 0.015 (− 0.035, 0.006)	0.155
HU	− 0.010 (− 0.015, − 0.006)	**< 0.001**	− 0.008 (− 0.013, − 0.003)	**0.002**
MPM	− 0.013 (− 0.256, 0.229)	0.915		

Bold indicates statistical significance

*ODI* Oswestry Disability Index; *CI* confidence interval; *BMI* body mass index; *PMI* psoas major muscle mass index; *HU* Hounsfield units; *MPM* morphological change in the psoas major

Patients with more severe back pain as assessed by the VAS score had a lower PMI (*p* < 0.001) and HU (*p* < 0.001) in the univariate analyses (Table 5). Multiple linear regression analysis revealed that the PMI was negatively correlated with back pain severity (*p* < 0.001). The PMI explained 40.8% of the variance in the VAS score for back pain.

**Table 5:** Association of patient characteristics and muscle parameters with back pain severity as measured with VAS

Subject characters	Univariable analysis		Multiple logistic regression	
	Coefficient, 95% CI	*p* value	Coefficient, 95% CI	*p* value
Gender	− 0.645 (− 1.556, 0.266)	0.163		
Age	− 0.020 (− 0.069, 0.029)	0.418		
BMI	0.003 (− 0.107, 0.113)	0.955		
Back pain duration	0.005 (− 0.0003, 0.011)	0.062		
Leg pain duration	0.002 (− 0.006, 0.010)	0.694		
Number of levels involved	0.147 (− 0.821, 1.116)	0.763		
PMI	− 0.793 (− 1.060, − 0.527)	**< 0.001**	− 0.666 (− 0.936, 0.368)	**< 0.001**
HU	− 0.158 (− 0.235, − 0.081)	**< 0.001**	− 0.075 (− 0.156, 0.005)	0.067
MPM	− 1.054 (− 5.173, 3.065)	0.613		

Bold indicates statistical significance

*VAS* visual analogue scale; *CI* confidence interval; *BMI* body mass index; *PM* psoas major muscle mass index; *HU* Hounsfield units; *MPM* morphological change in the psoas major

The patient characteristics and muscle parameters were not associated with leg pain severity as measured with the VAS score in the univariate analyses (Table 6).

**Table 6:** Association of patient characteristics and muscle parameters with leg pain severity as measured with VAS

Subject characters	Univariable analysis	
	Coefficient, 95% CI	*p* value
Gender	− 0.938 (− 2.009, 0.133)	0.085
Age	− 0.029 (− 0.081, 0.035)	0.424
BMI	0.023 (− 0.106, 0.153)	0.725
Back pain duration	0.003 (− 0.003, 0.010)	0.312
Leg pain duration	0.003 (− 0.006, 0.012)	0.510
Number of levels involved	− 0.316 (− 1.458, 0.826)	0.585
PMI	− 0.10 (− 0.463, 0.257)	0.572
HU	− 0.005 (− 0.103, 0.092)	0.914
MPM	− 0.005 (− 0.103, 0.092)	0.914

*VAS* visual analogue scale; *CI* confidence interval; *BMI* body mass index; *PMI* psoas major muscle mass index; *HU* Hounsfield units; *MPM* morphological change in the psoas major

## Discussion

To the best of our knowledge, this is the first study to analyze the association of the morphology of the psoas major muscle with the function and clinical symptoms in patients with SMLSS. Our main results showed that men had a higher PMI than women. Patients with severe disability had a lower HU value and PMI. Patients with moderate or severe back pain also had a lower HU value and PMI. The muscle attenuation and PMI were negatively correlated with the clinical ODI score and the VAS score for low back pain.

The fascicles of the psoas major muscle originate from the lumbar vertebral bodies, transverse processes, and intervertebral discs and insert into the femoral lesser trochanter [21]. The psoas major is widely known as a hip flexor and lumbar spine stabilizer through compression [22]. The CSA of the psoas major is mainly determined by the total number of muscle fibers [23]. The PMI and relative CSA (the CSA of the psoas major muscle divided by the CSA of the vertebral body or disc at the corresponding level) are the most commonly used morphological parameters to evaluate the psoas major muscle [10, 11, 16].

Fortin et al. [10] examined the relationship between the morphology of lumbar paraspinal muscles of individuals with L4/5 single-level LSS and functional status and symptoms. They discovered that the decrease of relative CSA of psoas (The muscle CSA divided by the CSA of the L5 vertebral endplate) was associated with the higher ODI and pain interference score. According to Chen et al. [11], the relative CSA of psoas was positively.

associated with functional status (evaluated by Japanese Orthopedic Association scores). Similar outcomes were founded in this study. We revealed that a higher PMI was associated with less severe back pain in univariable and multivariable analyses and with better functional status in univariable analyses. The possible reason is that all patients in this study had multilevel spinal stenosis, which is usually combined with extensive lumbar degeneration such as facet arthropathy and spondylosis [12]. These degenerated structures alter the normal biomechanics of the spine [24]. A higher PMI could play a stabilizing role for the lumbar spine, alleviating back pain due to instability [25, 26]. In our investigation, the results of univariate analysis revealed a positive link between PMI and functional status; however, multivariate analysis failed to detect this correlation, which might be caused by the limited sample size.

The psoas major muscle rarely undergoes fatty degeneration; therefore, few studies have evaluated the correlation between the psoas muscle density and lumbar degenerative diseases. Abbas et al. [27] discovered that the density of psoas major muscle [The value was obtained by calculating the mean density from measurements at three different locations (using a 50 mm^2^ circle)] in patients with LSS was higher than that in the control group; however, the author did not examine the relationship between the muscle density and functional status. Our results showed that although there was no obvious fat deposition in the psoas, there was still great variation in muscle density as assessed by the HU. The attenuation values of muscle on CT can provide quantitative data regarding the composition of muscle [28]. The attenuation value of muscle on CT was superior to muscle quantity in the evaluation of muscle function [29]. The ODI questionnaire was published in 1980 [30], and after several revisions it has become the most commonly used method to evaluate the functional status of patients with degenerative spinal disease [19]. Our results showed for the first time that higher muscle attenuation values were associated with a better functional status (lower ODI score). Like a higher PMI, higher muscle attenuation can reflect better muscle function, which is conducive to maintaining lumbar stability and in turn reducing the aggravation of symptoms and thus improving the functional status [25, 26].

The MPM can reflect muscle atrophy and may serve as a predictor of complications after colorectal cancer surgery [17]. There was no significant correlation of the MPM with the functional status or symptom severity of patients with SMLSS in the present study, suggesting that the long and short diameter of the psoas major may change in equal proportion in the pathogenesis of SMLSS.

There was also no correlation between the psoas major morphology characteristics and the VAS score for the lower extremities, indicating that the symptoms of the lower extremities in patients with SMLSS were caused by compression of lumbar nerve roots rather than paraspinal muscle degeneration [1].

Our study has several limitations. Firstly, because of its retrospective design, we were unable to control for accuracy in the documentation of medical treatment of low back pain or lower extremity pain before hospitalization, which may have affected the functional scores. Secondly, the muscle measurements were only obtained at the L3/4 intervertebral disc level using a single slice. Multiple-slice analysis and volumetric data may provide a more accurate muscle morphology assessment. Thirdly, the paraspinal muscles (mainly the psoas major, erector spinalis, and multifidus muscle) coordinate with each other to maintain the lumbar spine's movements and stability. This study only evaluated the relationship between the morphology of psoas major and the functional status and symptoms. In the future, it is necessary to comprehensively evaluate the clinical effect of morphology and strength of paraspinal muscles. Finally, this study had a relatively small sample size and was conducted at a single center. Further prospective multicenter studies are needed to confirm our findings.

## Conclusions

This study suggests that the muscle attenuation and PMI of the psoas major are closely related to the functional status and severity of back pain in patients with SMLSS. Considering the high perioperative risk of surgical treatment for SMLSS, development of safe and effective conservative treatment is of great clinical significance. Therefore, whether improvement in the quality and quantity of the psoas major muscle through exercise can alleviate the clinical symptoms and improve the functional status of patients with SMLSS should be examined in future studies.

## Data Availability

The datasets generated and analyzed during the current study are available from the corresponding author upon reasonable request.
